# The bZIP Protein MeaB Mediates Virulence Attributes in *Aspergillus flavus*

**DOI:** 10.1371/journal.pone.0074030

**Published:** 2013-09-09

**Authors:** Saori Amaike, Katharyn J. Affeldt, Wen-Bing Yin, Stephen Franke, Anjali Choithani, Nancy P. Keller

**Affiliations:** 1 Department of Plant Pathology, University of Wisconsin-Madison, Madison, Wisconsin, United States of America; 2 Department of Medical Microbiology and Immunology, University of Wisconsin-Madison, Madison, Wisconsin, United States of America; 3 Department of Bacteriology, University of Wisconsin-Madison, Madison, Wisconsin, United States of America; University of Nebraska, United States of America

## Abstract

LaeA is a fungal specific virulence factor of both plant and human pathogenic fungi. Transcriptional profiles of *laeA* mutants have been successfully exploited to identify regulatory mechanisms of secondary metabolism in fungi; here we use *laeA* mutants as tools to elucidate virulence attributes in *Aspergillus flavus*. Microarray expression profiles of Δ*laeA* and over-expression *laeA* (*OE::laeA*) were compared to wild type *A. flavus.* Strikingly, several nitrogen metabolism genes are oppositely mis-regulated in the Δ*laeA* and *OE::laeA* mutants. One of the nitrogen regulatory genes, the bZIP encoding *meaB,* is up-regulated in Δ*laeA*. Significantly, over-expression of *meaB* (*OE::meaB*) phenocopies the decreased virulence attributes of a Δ*laeA* phenotype including decreased colonization of host seed, reduced lipase activity and loss of aflatoxin B1 production in seed. However, a double knock-down of *laeA* and *meaB* (*KD::laeA,meaB*) demonstrated that *KD::laeA,meaB* closely resembled Δ*laeA* rather than wild type or Δ*meaB* in growth, aflatoxin biosynthesis and sclerotia production thus suggesting that *meaB* does not contribute to the Δ*laeA* phenotype. MeaB and LaeA appear to be part of regulatory networks that allow them to have both shared and distinct roles in fungal biology.

## Introduction

*Aspergillus flavus* is an opportunistic phytopathogen that colonizes oil-rich seeds such as maize, peanuts, and treenuts before and after harvest [1], [2]. This fungus is infamous for the production of polyketide-derived carcinogenic and mutagenic secondary metabolites known as aflatoxins. Aflatoxins cause aflatoxicosis resulting from ingesting high levels of aflatoxin-contaminated food or feed, and long-term exposure can lead to liver carcinoma [1], [3]. There are few pre-harvest controls, but successful post-harvest measures deployed to control *A. flavus* and aflatoxin contamination include controlling temperature and moisture levels of stored grain. Control in pre-harvest venues requires an understanding of *A. flavus* biology and fungal pathways required for success as a pathogen. Currently there is little understanding of the molecular mechanisms involved in *A. flavus* infection of host tissue. One of the few proteins found to govern virulence in *A. flavus* is LaeA, as well as its interactor, VeA [4].

In previous research LaeA and VeA were found to regulate developmental processes in *A. flavus* including sclerotial, conidial and aflatoxin production [4]–[6]. LaeA is a global regulator of secondary metabolism in filamentous fungi, including *Aspergillus, Penicillium*, *Fusarium* and *Cochliobolus* species [7]–[10]. LaeA is located in the nucleus where it partners with VeA and another protein called VelB to form what is known as the Velvet Complex [11]. VeA is a light regulated protein controlling spore development and secondary metabolism [12]. Both VeA and LaeA have been found to be pathogenicity factors in the plant pathogens *A. flavus*, *F. fujikuroi*, *F. graminearum* and *C. heterostrophus* [4], [6], [8], [10], [13] and LaeA in invasive aspergillosis in the human pathogen *A. fumigatus* [7].

The central role of LaeA in secondary metabolite synthesis has lent itself well to using LaeA mutants as tools to uncover mechanisms in their regulation [14], [15]. For instance, one recent study identified a novel bZIP transcriptional factor termed RsmA (restorer of secondary metabolism A) that partially restored sterigmatocystin in both Δ*laeA* and Δ*veA* mutants in *A. nidulans* [16], [17]. bZIP proteins are eukaryotic transcription factors well described in yeast and, increasingly, in filamentous fungi [18], [19]. The identification of RsmA through the Δ*laeA* mutagenesis screen served to emphasize the recently recognized role of bZIP proteins in fungal secondary metabolism. For example, the bZIPs AtfB and Apyap1 have been reported to positively and negatively regulate aflatoxin in *A. parasiticus* respectively [20], [21], Aoyap1 negatively regulates ochratoxin in *A*. *ochraceus* [22], and MeaB negatively regulates bikaverin in a nitrogen dependent manner in *F. fujikuroi* [23]. MeaB has also been found important in transmitting a conserved nitrogen-responsive pathway to control infectious growth in the vascular wilt pathogen, *F. oxysporum* [24].

Considering the usefulness of examining LaeA mutants in elucidating cellular processes governing secondary metabolism in fungi, it seemed that LaeA could also serve as a tool to uncover virulence mechanisms in fungi. A previous transcriptional profiling study aimed at viewing global secondary metabolism regulation in *A. flavus* utilized both Δ*laeA* and *OE::laeA* mutants to examine expression of this species’ 55 secondary metabolite gene clusters [15]. We hypothesized that this profiling data could also provide clues to *A. flavus* virulence pathways as the Δ*laeA* strain is reduced in its ability to colonize host seed [4], [6]. Re-examination of the array set showed that several GO categories other than secondary metabolism are regulated by LaeA, including many genes involved in nitrogen metabolism. In particular, the nitrogen regulatory bZIP gene, *meaB,* is up-regulated in the Δ*laeA* background. We examined both *meaB* over-expression and deletion strains for pathogenicity attributes. Whereas loss of *meaB* did not affect seed pathogenesis, the *OE::meaB* strain phenocopied several hypovirulent Δ*laeA* traits including impairment in seed colonization, lipase activity and aflatoxin production in seed. However, a strain with both *laeA* and *meaB* depleted by RNAi does not restore wild type phenotype but resembles Δ*laeA* for growth, aflatoxin biosynthesis and sclerotia production.

## Results

### Nitrate Metabolic Genes are Mis-regulated in laeA Mutants

**Figure 1:**
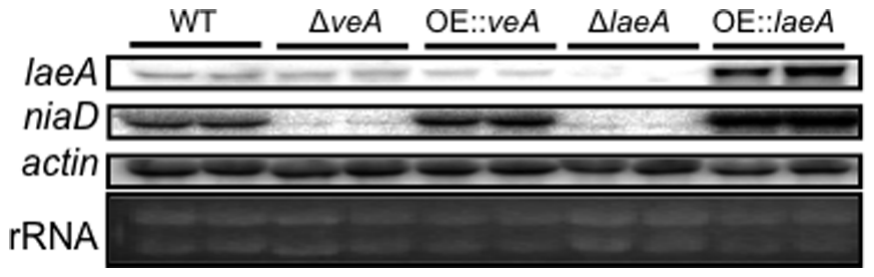
Northern analysis of *Aspergillus flavus* velvet complex mutants. VeA and LaeA mutants [4] grown in liquid GMM conditions under dark for 48 hours at 250 rpm. Note increase and decrease of *niaD* expression in the *laeA* deletion (Δ*laeA* TJW71.1) and over-expression (*OE::laeA* TJW79.13) respectively. Δ*veA* (TSA1.54) also shows decreased *niaD* expression. *OE::veA* = TSA2.46.

Georgianna *et al*. [15] examined 28 diverse conditions for the regulation of 55 secondary metabolite gene clusters in *A. flavus*. Nine of these conditions compared transcriptional profiles of wild type, *ΔlaeA* and *OE::laeA* strains grown in three different environments (liquid shake at 6 and 24 hours or stationary growth at 24 hours). Not unexpectedly, LaeA was found to be a positive regulator of many of the secondary metabolite clusters including aflatoxin. Re-examination of this data showed that several other GO categories were highly regulated by LaeA. Notably, some genes involved in nitrate utilization were oppositely expressed in the Δ*laeA* and *OE::laeA* strains. In general, nitrate metabolism genes were down regulated in Δ*laeA* but up-regulated in *OE::laeA* (Table 1), this was confirmed for *niaD* by northern analysis (Figure 1). A decrease in *niaD* expression was also observed in the *ΔveA* mutant (Figure 1). Additionally, one of the negative nitrogen regulatory proteins previously characterized in fungi, MeaB, was up-regulated in the Δ*laeA* strain under sclerotial induction conditions (Table 1). Expression of two other global nitrogen regulators, *nmrA* and *areA*, were not statistically different in these conditions.

**Table 1:** Expression of nitrogen metabolism genes in comparisons of wild type to either Δ*laeA* or *OE::laeA* strains.

			Δ*laeA*	*OE::laeA*	Δ*laeA*	*OE::laeA*	Δ*laeA*	*OE::laeA*
Accession #		Gene	Sclerotia		6 hr Liquid		24 hr Liquid	
AFLA_018790	AFL2G_03510.2	*crnA*	–	+	NS	NS	NS	NS
AFLA_018800	AFL2G_03511.2	*niiA*	–	+	–	NS	–	+
AFLA_018810	AFL2G_03512.2	*niaD*	–	+	–	+	–	+
AFLA_049870	AFL2G_10206.2	*areA*	NS	NS	NS	NS	NS	NS
AFLA_005620	AFL2G_09875.2	*nmrA*	NS	NS	NS	NS	NS	NS
AFLA_031790	AFL2G_03512.2	*meaB*	+	NS	NS	NS	NS	NS

Data summarized from [15].

− = decreased expression in *laeA* mutant versus wild type.

+ = increased expression in *laeA* mutant versus wild type.

NS = not significant difference in expression in *laeA* mutant versus wild type.

*crnA*: nitrate transporter, *niiA*: nitrite reductase, *niaD*: nitrate reductase, *areA*: GATA transcriptional activator, *nmrA*: nitrogen metabolite repression regulator, *meaB*: methylammonium resistant B.

### Creation of meaB Mutants

Both *meaB* deletion and over-expression (OE) strains were created through standard transformation processes. Twenty transformants were obtained from transformation of the *A. flavus OE::meaB* allele and two transformants, TSA15.13 and 15.18, were found correct using PCR, sequencing, Southern, and Northern analysis (Figure S1A). The *A. flavus meaB* allele was replaced with either the *A. fumigatus pyrG* allele (2 correct strains, TSA14.13 and 14.18, out of twenty transformants, Figure S1B) or the *A. parasiticus pyrG* allele (3 correct strains, TSA19.4, 19.7, and 19.8, out of 20 transformants, Figure S1C). Both *pyrG* alleles were used as replacement markers as we found the *A. fumigatus pyrG* replacements required uracil and uridine supplementation for normal growth (Figure S2). However, the same was found to be true for the *A. parasiticus pyrG* replacement strains (data not shown).

These results could indicate a mutation in both *pyrG* genes, a marker gene effect (e.g. *pyrG* is not expressed to full levels at this locus), or that loss of *meaB* affects pyrimidine metabolism. To determine which of these possibilities was most likely, both *pyrG* replacement genes were sequenced and found to be intact. Next, knock-down (KD) *meaB* mutants were created using RNA silencing technology and one representative transformant named TSA23.15 was assessed (Figure S1D). The KD strain also required uracil and uridine supplementation for normal growth despite placement of *pyrG* at a different locus (Figure S2). The *OE::meaB* strain, using the same marker gene, did not required uracil and uridine supplementation. To account for the supplementation needed by the *meaB* deletion strains, all growth media experiments were carried out with uracil and uridine supplementation. The mutant strains chosen for the following studies were TSA15.18 (*OE::meaB*) and TSA14.13 (Δ*meaB*).

### meaB Affects A. Flavus Virulence

MeaB has been reported as critical in transmitting nitrogen signaling through a MAPK cascade in controlling infectious growth of the vascular wilt pathogen *F*. *oxysporum* [24]. Specifically, deletion of *meaB* in this fungus allowed fungal invasion of host tissue during ammonium repressive conditions when normally the fungus is unable to colonize host tissue, hence presenting an enhanced aggressiveness in this particular environment. Here, virulence of *A. flavus meaB* mutants were tested directly by assessing growth on peanut seed and indirectly through observation of lipase activity, a strong indicator of degradative powers required for seed invasion.

**Figure 2:**
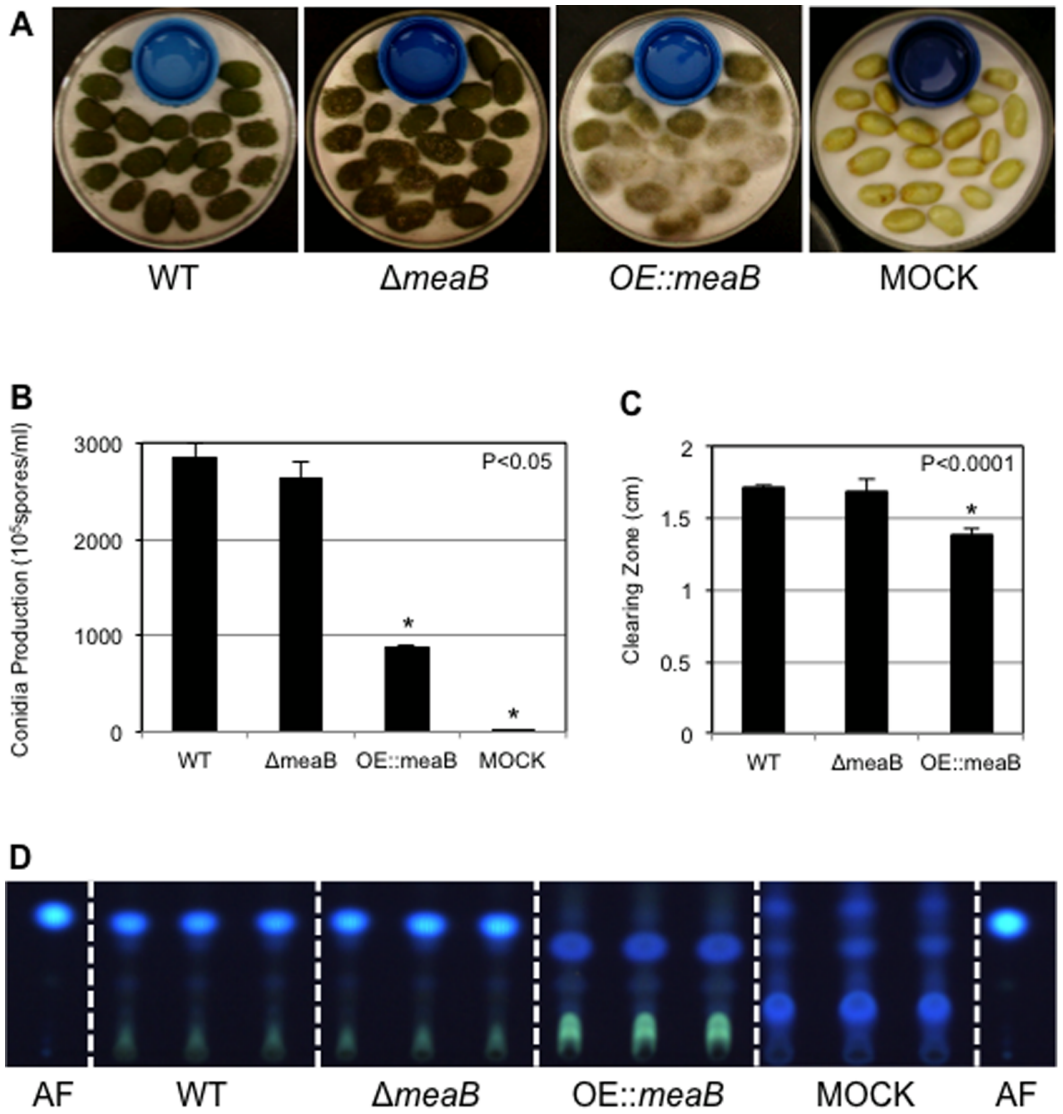
Pathogenicity of *A. flavus meaB* mutants. (A) Growth of fungal colonies on living peanut cotyledons after 5 days of inoculation. (B) Conidia production on peanut cotyledons after 5 days of inoculation. Asterisk indicates statistical significance at P<0.05. (C) Lipase activity of *meaB* mutants. Asterisk indicates statistical significance at P<0.0001. (D) Thin layer chromotrography measurements of aflatoxin B1 extracted from seed in Panel A. AF = aflatoxin B1 standard.

As shown in Figure 2A and 2B, the *OE::meaB* strain was crippled in its ability to colonize and sporulate on host seed. Although the Δ*meaB* strains showed some impairment in growth media without supplemented uracil and uridine (Figure S2), they grew equally well on seed as wild type, thus suggesting all nutritional needs of the Δ*meaB* strain were met by the host seed. At 3 days, Δ*meaB* and wild type strains started to conidiate on the seed surface (data not shown). At 5 days, these strains had colonized the entire surface of the seed whereas the over expression strain was delayed in conidiation and, moreover, grew aerially with fluffy mycelia (Figure 2A). These visual results were reflected by conidial counts in which sporulation was significantly decreased in the *OE::meaB* strain (Figure 2B).

The poor seed colonization by *OE::meaB* was similar to that described for the Δ*laeA* mutant [4]. We also assessed two other parameters of seed infection impacted by *laeA* loss. First, we assessed the ability of the strain to degrade the lipase substrate glyceryl tributyrate and found, like the Δ*laeA* mutant, *OE::meaB* showed significantly less lipase activity than either wild type or the Δ*meaB* strains (Figure 2C). Finally we assessed aflatoxin B1 production in the infected seed and observed a visual loss of B1 production by this strain (Figure 2D). Both the decreased lipase activity and loss of aflatoxin B1 production recapitulated that of the Δ*laeA* strain.

### Depletion of meaB in a laeA Knockdown Background does not Restore Aflatoxin or Sclerotial Production

The virulence data supported a view that LaeA may indeed act through MeaB. To more thoroughly address this hypothesis, we generated knock-down mutants in which both *laeA* and *meaB* were depleted by RNAi technology (*KD::laeA,meaB*). Nine transformants were confirmed to be correct by Northern and Southern analysis (Figure S3). RNAi mutants can display different phenotypes depending on the level of gene repression, so of the nine that were correct, we selected four for subsequent experiments (TKJA20.1, 20.4, 20.8, and 20.12). These strains and their controls were assessed for their ability to produce aflatoxin and sclerotia with the thought that both parameters could be restored in the KD strains.

**Figure 3:**
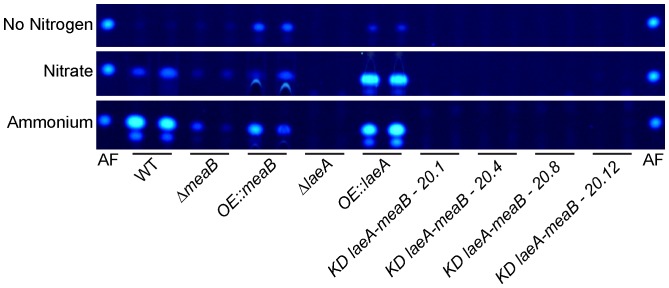
Aflatoxin production on different nitrogen sources. The indicated strains were grown in GMM lacking nitrogen, standard GMM (70.6 mM sodium nitrate), or GMM with 70.6 mM ammonium chloride for two days as described in Materials and Methods. All media was supplemented with uracil and uridine. 40% of extracted metabolites were loaded onto the TLC plates except in the cases of wild type on GMM with ammonium, as well as *OE::laeA* on GMM with nitrate and GMM with ammonium. For these three samples, 10% of extracted metabolites were loaded because of the high levels of AF biosynthesis. AF = aflatoxin standard.

Aflatoxin synthesis in *A. flavus* has been linked to nitrogen metabolism as nitrate medium is generally regarded as a poor substrate for aflatoxin synthesis [25]–[29]. In contrast, ammonium as nitrogen source is reported to support high levels of aflatoxin production [28], [29]. Therefore, to more thoroughly investigate the possibility that nitrogen source, potentially mediated by MeaB, could differentially regulate aflatoxin synthesis, we examined aflatoxin production on both nitrate and ammonium medium. Figure 3 confirms that wild type produced low levels of aflatoxin on nitrate medium and high levels of aflatoxin on ammonium medium. The *OE::laeA* strain produced high levels of aflatoxin regardless of nitrogen source and, as noted previously, Δ*laeA* strain did produce aflatoxin. Despite not producing aflatoxin in seed, the *OE::meaB* strain produced aflatoxin on growth media, in a pattern similar to *OE::laeA*. The Δ*meaB* mutant produced little aflatoxin, and all four isolates of *KD::laeA,meaB* even less, similar to Δ*laeA*.

**Figure 4:**
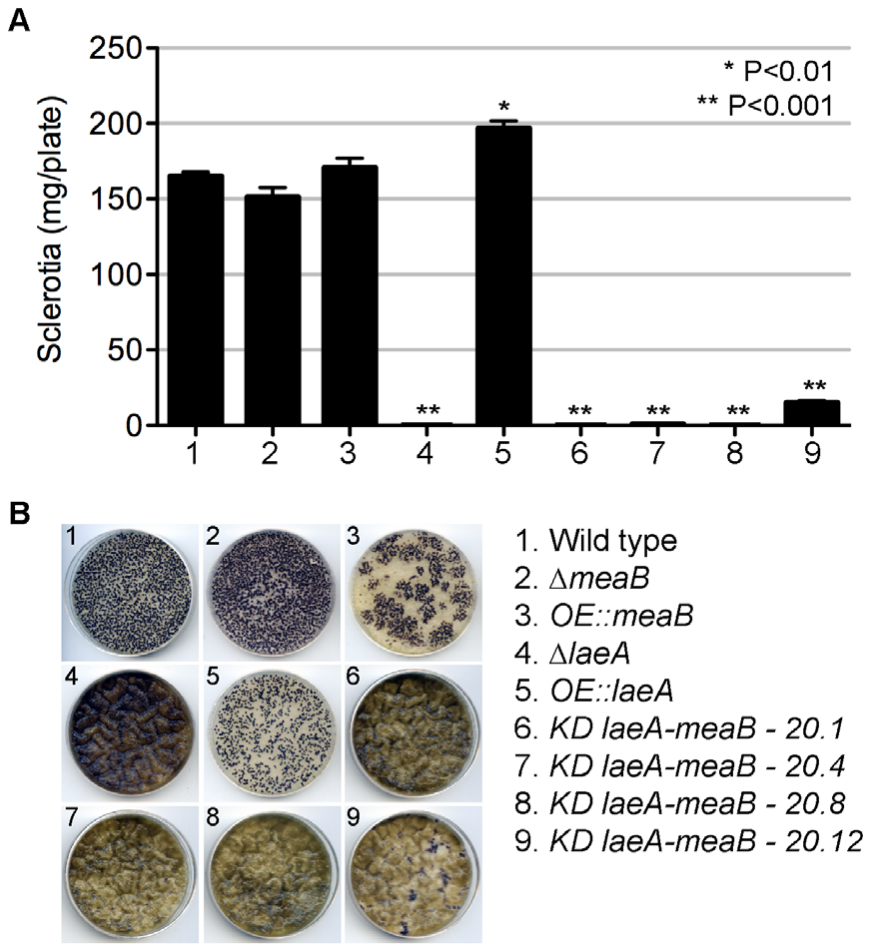
Sclerotia production of *meaB* mutants. (A) The strains listed were grown on GMM plus 2% sorbitol to induce sclerotia production. All media was supplemented with uracil and uridine. Asterisks indicate significant differences between each strain relative to the wild type as determined by a Student T test, with * = P<0.01 and ** = P<0.001. (B) One representative plate for each strain is shown here before removal of sclerotia. Despite less coverage on the plate, the *OE::laeA* sclerotia were of greater mass than the other strains.

In addition to repressing aflatoxin biosynthesis, another striking phenotype of *laeA* loss is the concomitant loss of the overwintering bodies called sclerotia [4], [6]. However, neither Δ*meaB* nor *OE::meaB* strains displayed statistically significant loss or over production of sclerotia compared to wild type (Figure 4), although the *OE::meaB* mutant displayed unusual distribution of sclerotia on the plate. Of the four isolates of *KD::laeA,meaB*, two (TKJA20.1 and 20.8) made no sclerotia, one (TKJA20.4) had several per plate, and one (TKJA20.12) produced 9% of the wild type sclerotial mass. Thus these strains displayed a similar sclerotial phenotype as Δ*laeA*.

### meaB Deletion but not KD::laeA,meaB Strains are Affected in Nitrogen Utilization

**Figure 5:**
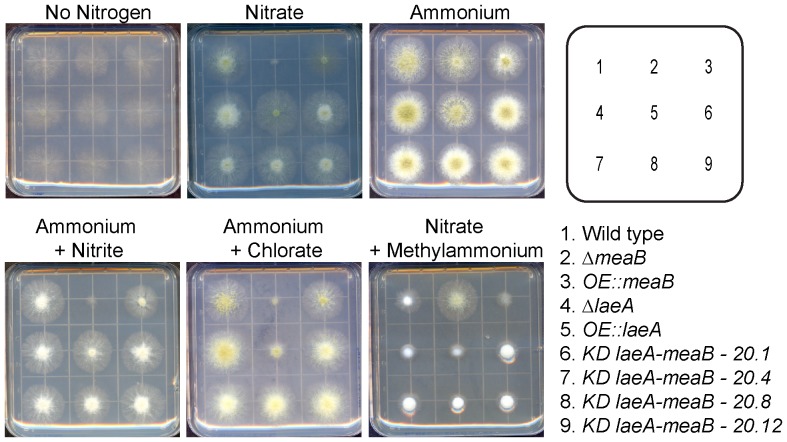
Growth of *A. flavus meaB* mutants on different nitrogen sources. Plates containing GMM with no nitrogen, GMM with 10 mM sodium nitrate, GMM with 10 mM ammonium chloride, GMM with 10 mM ammonium chloride plus 30 mM sodium nitrite, GMM with 10 mM ammonium chloride plus 200 mM potassium chlorate, or GMM with 10 mM sodium nitrate plus 100 mM methylammonium chloride were inoculated with the indicated strains of *A. flavus* and grown for 3 days at 29°C. All media was supplemented with uracil and uridine.

Because our results from the KD strains did not support the hypothesis that the Δ*laeA* phenotype was mediated by MeaB over-expression, we were curious if this non-remediation would also be exhibited on growth on nitrogen sources and toxic analogs [23], [30]. The *meaB* deletion phenotype was the same as those described earlier for *A. nidulans* and *F. fujikuroi* mutants where *meaB* loss resulted in repressed growth on medium amended with the toxic nitrate analog chlorate but enhanced growth on methyl ammonium medium (Figure 5). Growth of the Δ*meaB* strain was also restricted on nitrate and nitrite media. As predicted, the *OE::meaB* strain showed enhanced growth over Δ*meaB* on chlorate medium but a near inability to grow on methylammonium medium. Both *OE::laeA* and Δ*laeA* strains grew nearly equivalent to wild type on all media tested, except for *OE::laeA* which exhibited repressed growth on chlorate medium. All four isolates of *KD::laeA,meaB* exhibited similar growth patterns to those of the Δ*laeA* mutant and not the Δ*meaB* mutant on the different nitrogen sources.

## Discussion

The *meaB* gene was described in 1996 in a study looking for genes involved in nitrogen metabolite repression in *A. nidulans* [31]. The protein was further defined as a regulatory factor in *A. nidulans* where it was proposed to activate NmrA [30], a repressor of the GATA factor AreA known to regulate several genes required for nitrate utilization [32], [33]. From these studies both MeaB and NmrA were regarded as repressive nitrogen regulatory proteins. Another study, however, has recently shown *nmrA* expression in *A. nidulans* and *F. fujikuroi* is not *meaB* dependent [23]. All three proteins, MeaB, NmrA and AreA are conserved in filamentous fungi, and recent studies with MeaB have implicated a role for this protein not only in nitrogen metabolism but also secondary metabolism (e.g. bikaverin production, [23]) and regulation of virulence cascades [24] in *Fusarium* species. The finding that *meaB* and nitrate metabolism genes were regulated in *A. flavus* by *laeA* in a microarray study [15], a conserved virulence factor in pathogenic fungi [4], [6], [8], [10], [13], led us to ask if any aspects of virulence in *A. flavus* were mediated by MeaB. Specifically *meaB* expression was significantly upregulated in the *A. flavus* Δ*laeA* strain leading us to hypothesize that *OE::meaB* might be responsible, to some extent, for the Δ*laeA* phenotype.

Deletion of *laeA* leads to several striking phenotypes contributing to decreased virulence on host seed including decreased colonization as reflected by lower conidial production, an impairment in lipase activity and, finally, loss of aflatoxin production on seed [4], [6]. All three of these characteristics were also displayed by the *OE::meaB* mutant (Figure 2). This data suggested that *meaB* over-expression in the *ΔlaeA* strain might contribute to the decreased virulence of the Δ*laeA* strain. However, our characterization of the *KD::laeA,meaB* strain argues against this hypothesis, as the *KD::laeA,meaB* strains closely mimic the Δ*laeA* phenotype and not the Δ*meaB* or wild type phenotypes for nitrogen utilization and ability to produce sclerotia and aflatoxin. This implies that, despite the connection by microarray, the roles of LaeA and MeaB are distinct in *A. flavus*.

The only other study assessing *meaB* impact on pathogenicity is with the tomato pathogen *F. oxysporum* in which a Δ*meaB,* but not an *OE::meaB,* strain was assessed for a role in virulence [24]. In the *F. oxysporum* study, loss of *meaB* resulted in increased virulence in ammonium supplemented pathogenicity assays. The authors of that study suggested that MeaB normally inhibits SteA activation of a MAPK signaling pathway in *F. oxysporum*, and that loss of this inhibition could explain the enhanced virulence of the Δ*meaB* strain. Whereas there was no evidence of increased virulence of the *A. flavus* Δ*meaB* strain, the *OE::meaB* strain was reduced in virulence. We found an opposite regulation of *steA* by *meaB* in *A. flavus* (Figure S4) suggesting that the impact of *meaB* on virulence in *A. flavus* does not work through the same signaling pathways as *F. oxysporum.* However, it is possible that *steA* over-expression governed some of the *OE::meaB* phenotypes described in this study. SteA has been characterized in several *Aspergillus spp.* In *A. oryzae* [34], a species now considered as a clade of *A. flavus*, over-expression of *steA* led to restricted vegetative growth in *A. oryzae* and may explain, in part, the somewhat restricted vegetative growth of the *A. flavus OE::meaB* strain on media. In *A. nidulans steA* is required for sexual development [35] and while we observed that the *A. flavus* strain *OE::meaB* produced equivalent mass of sclerotia (the analogous *A. flavus* structure to cleistothecia) to wild type, the sclerotia appeared larger and were clumped together on the plate instead of evenly distributed like wild type (Figure 4). Further suggesting that MeaB may be involved in sclerotia formation, two of the four *KD::laeA,meaB* isolates were able to restore a small amount of sclerotia in comparison to the complete loss of sclerotia in Δ*laeA*. However, it is not known whether this is due to different levels of gene repression – of either *laeA* or *meaB* - or varying off-target effects among the isolates.

The requirement for uracil and uridine supplementation in the *ΔmeaB* and *KD::meaB* strains was of note. A position effect for *pyrG* function has been previously described [36], [37] and may explain the observations in this study. However, because the *pyrG* gene located to a different position in the KD strain than in the deletion strains, yet the KD mutant still required supplementation and the *OE::meaB* strain (where *pyrG* located to the same region as in the *ΔmeaB* strains) did not require supplementation, it is possible that MeaB could be involved in pyrimidine metabolism. Although there is no report for a requirement for MeaB in pyrimidine synthesis in the literature, another nitrogen global regulator, AreA, has been connected with pyrimidine metabolism [38]. Whatever the mechanism underlying the requirement for supplementation in growth medium, there was no observable impact on growth of the deletion strain on seed.

In summary, through LaeA microarray sleuthing, we have identified MeaB as an important controller of *A. flavus* virulence and toxin attributes including seed colonization and aflatoxin synthesis on seed. However, it appears that *meaB* plays little – if any – role in the pleiotropic effects of *laeA* loss on fungal biology as LaeA and MeaB exhibit distinct roles in growth and development. This finding is in contrast to another study where a LaeA regulated gene, *nosA*, was found to mediate the decreased radial growth and delayed conidial germination observed in *A. fumigatus ΔlaeA* [39]. Considering that upwards of 10% of fungal genome is regulated by LaeA, it is likely that only a subset of the LaeA regulated genes would impact the *ΔlaeA* phenotype when deleted or overexpressed in this background.

## Materials and Methods

### Microarray Data

The microarray data used for this study has been published and is deposited in the Gene Expression Omnibus (GEO) database under accession number GSE15435 [15].

### Fungal Strains and Growth Conditions

*Aspergillus flavus* strains used and created in this study are listed in Table 2. All strains were maintained as glycerol stocks and grown on glucose minimal media (GMM, [40]) amended with appropriate supplements for *A. flavus* spore production.

**Table 2:** Fungal strains and plasmids used in this study.

strains	genotype	References
*Aspergillus flavus*		
NRRL3357	Wild type	[45]
NRRL3357.5	*pyrG-*	[45]
TSA 1.54	Δ*veA*::*A. fum pyrG*	[4]
TSA 2.46	*A. fum pyrG*, *veA*	[4]
TJW 71.1	Δ*laeA*::*A. fum pyrG*	[6]
TJW79.13	Δ*laeA*:: *A.fum pyrG*, *niaD*-, *niaD*, *laeA*	[6]
TSA 2.8	*A. fum pyrG*, *veA*, *niaD*, *laeA*	[4]
TSA14.13	*ΔmeaB::A. fum pyrG*	This study
TSA15.18	*A. fum pyrG::A. nidulans gpdA(p):: meaB*	This study
TSA19.4	*ΔmeaB::A. parasiticus pyrG*	This study
TSA23.15	*pyrG-*, IRT *meaB*, *A. fumigatus pyrG*	This study
TKJA20.1	*pyrG-*, IRT *meaB laeA, A. fumigatus pyrG*	This study
TKJA20.4	*pyrG-*, IRT *meaB laeA, A. fumigatus pyrG*	This study
TKJA20.8	*pyrG-*, IRT *meaB laeA, A. fumigatus pyrG*	This study
TKJA20.12	*pyrG-*, IRT *meaB laeA, A. fumigatus pyrG*	This study
plasmid		
pJW24	*A. parasiticus pyrG*	[46]
pTMH44.2	*gpdA(p)::gfp::trpC(t)*	[47]
pJW66.3	*gpdA(p)::ppoA,ppoB,ppoC::gfp::ppoC,ppoB,ppoA::trpC(t), A. fum pyrG*	[43]
pSA17.3	*gpdA(p)::meaB::gfp::meaB::trpC(t)*	This study
pKJA38.6	*gpdA(p)::meaB,laeA::gfp::trpC(t)*	This study
pKJA39.1	*gpdA(p)::meaB,laeA::gfp::laeA,meaB::trpC(t)*	This study
pKJA40.1	*gpdA(p)::meaB,laeA::gfp::laeA,meaB::trpC(t), A. fum. pyrG*	This study

*A. fum = A. fumigatus.*

### Creation of Fungal Strains

#### *Aspergillus flavus meaB* over-expression and deletion constructs

An over-expression *A. flavus meaB* (AFLA_031790, also called AFL2G_02367.2) cassette was created by using the primers listed in Table 3. To create *meaB* over-expression cassettes, 4 PCR fragments were created and fused in this order: 1.5 kb of *meaB* upstream fragment, 1.97 kb *A. fumigatus pyrG,* 1.5 kb *A. nidulans gpdA* promoter, and 1.5 kb *meaB* open reading frame. First, the *A. fumigatus pyrG* and *A. nidulans gpdA* PCR fragments were fused and gpdA(p)::pyrGFor and *A. nidulans* gpdA(p)Rev primers for the *gpdA* fragment. Next, the *meaB* upstream flank, OEAFmeaB5FFor and OEAFmeaB5FRev, and the *meaB* open reading frame, OEAFmeaB3FFor and OEAFmeaB3FRev, PCR fragments were fused to the *A. fumigatus pyrG*::*A.nidulans gpdA* promoter PCR fragment. The final construct was confirmed with restriction endonuclease and sequencing.

**Table 3:** Oligonucleotides used in this study.

Primer	Sequence	
*A. nidulans* gpdA(p)For	AAG GCT TG GGC CGC TGC GTT GGT T	
gpdA(p)::pyrG For	GTG ACG ACA ATA CCT CCC GAC ACC TGG CAT CCG GAT GTC GAA GGC TTG	
*A. nidulans* gpdA(p) Rev	CAT GGT GAT GTC TGC TCA AG	
*A. fumigatus pyrG* For	TGCCTCAAACAATGCTCTTC	
*A. fumigatus pyrG* Rev	CAAGGTATCGTCGGGAGGT	
ANOEmeaB5FFor	CCTTCTTCTGCCATGACATCGG	
ANOEmeaB5FRev	AAGACACCTTTGCGCAGTGGTGGCTTGGGGCACCTGCGTTGG	
ANOEmeaB3FFor	GAGGGTGAAGAGCATTGTTTGAGGCA CTTTCGATCCGTTTCCGTCGATG	
ANOEmeaB3FRev	GAGTTAGACCCCAACAGAGAGC	
OEAFmeaB5FFor	CCAGGCCAGTATGATTTCCCAG	
OEAFmeaB5FRev	GATATACTTCCCCTCAAATAGAATCCACCAGGTATCGTCGGGAGGTATT	
OEAFmeaB3FFor	AGCATGCACTTCCAGACCCCCTTCAGCTACGAACAGAATAATACTCGTCACTAA	
OEAFmeaB3FRev	CTTAGGTGGTGAAGGGAGGG	
KOAFmeaB3FFor	GAAGAGGGTGAAGAGCATTGTTTGAGGCAACACCACTTTCCGCTCTCTACTC	
KOAFmeaB3FRev	GATAGTCTCTTCTACTTTTCTGGC	
OEAFmeaB5FnestedFor	GGATCGGCATTAGTTCGAACGG	
KOAFmeaB3FnestedRev	CGCTCCTCCCACAAAAGCTATC	
KOAFmeaB5FRev AP	GATGATAAGCTGTCAAACATGAGTGGATTCTATTTGAGGGGAAGTATATC	
KOAFmeaB3FFor AP	CCACACCCGTCCTGTGGATCACACCACTTTCCGCTCTCTACTCTCGGTC	
pTMH44seq1For	CTACATCCATACTCCATCCTTC	
pTMH44seq1rev	GTGGCCGAGAATGTTTCCATCC	
pTMH44seq2For	CAAGTTCGAAGGTGACACCCTG	
pTMH44seq2Rev	CCATTTGTCTCAACTCCGGAGC	
gpdA(p) intFor scr	GTTGACAAGGTCGTTGCGTCAG	
NAFnmrA For scr	CAACAGAAGACCATTGCCGTCG	
NAFnmrA Rev scr	CAACATCATGCTCCGCATCCAAC	
NAFlaeA For	CCTTGTATGATGTATGTATGATGAGC	
NAFlaeA Rev	GACAGCGAAAGTGAAGAGGACATC	
NAFactin For	GAAGCGGTCTGAATCTCCTG	
NAFactin Rev	ACAGTCCAAGCGTGGTATCC	
NAFniaD For	AGAGATCTCGACGAGAGACCTG	
NAFniaD Rev	GGTCTCTAAGCATCACCATCCC	
NAFsteA For	CTCGGGTACCGATATTGTTCGG	
NAFsteA Rev	GAAATGAGCGCTTGCGAGATTTG	
KS H-N-laeA F	TATAAAGCTTCCATGGAGGAGTTCTGAAAAACAGCC	
KS laeA-meaB R	ATTCCAAACGGCTCTCCTTTGGGTGCGATCAGGACTCTCGAAGTCGAATG	
KS laeA-meaB F	TTTTACGCCCCATTCGACTTCGAGAGTCCTGATCGCACCCAAAGGAGAGC	
KS meaB-A-B R	TATAGGATCCGGCGCGCCACGACTCATGCTCCTCATCG	
KS laeA int F	CAGACGCTTTCGTTGTTGGG	
KS laeA int R	TTGAACGCCTCCGACTTGAC	
KS meaB int F	GAAGCAACGTAACCGTCAGG	
KS meaB int R	ACGACTCATGCTCCTCATCG	
KS JP-M13 F	GTAAAACGACGGCCAGTG	
KS JP-M13 R	GGAAACAGCTATGACCATG	

*Aspergillus flavus meaB* deletion cassettes were created where *meaB* was replaced with *A. fumigatus pyrG* or *A. parasiticus pyrG*. These were created by PCR amplifying 1.5 kb of the *meaB* upstream fragment from NRRL3357 gDNA, 1.97 kb *A. fumigatus pyrG* from AF293 gDNA or 3 kb *A. parasiticus pyrG* from pJW24 plasmid, and 1.5 kb of the *meaB* downstream fragment from NRRL3357 gDNA using the following primers: OEAFmeaB5FFor and OEAFmeaB5FRev or KOAFmeaB5FRevAP, *A.parasiticus pyrG*For and Rev, *A.fumigatus pyrG*For and Rev, KOAFmeaB3FFor or KOAFmeaB3FForAP and Rev. Three PCR fragments were fused as previously described [41]. The final constructs were confirmed with restriction endonuclease and then transformed into *A. flavus* NRRL 3357.5.

#### RNAi constructs

A RNAi silencing construct to down regulate *meaB* expression in *A. flavus* was generated in the following manner. First, a 498 bp PCR fragment of *A. flavus meaB* was amplified from wild type gDNA using AFKDmeaB5F For and 5FRev, and this was then inserted into the *Nco*I and *Asc*I site of pTMH44.2 through quick-change method [42] to create pSA16.5. After confirming the sequence and direction of the *meaB* insert using PCR with primers with pTMH44seq1For and seq1Rev, the same PCR fragment was amplified with AFKDmeaB3F For and Rev from wild type gDNA and ligated into the *Not*I and *Bam*HI site of pSA16.5 to create pSA17.3. This transformation vector was confirmed by PCR with primers pTMH44seq1For and seq2Rev, endonuclease digestion, and sequencing using pTMH44seq2 For and seq2Rev.

RNAi technology was also used to create a strain with both *laeA* and *meaB* transcripts depleted. A 342 bp fragment of *A. flavus laeA* was amplified with primers KS H-N-laeA F and KS laeA-meaB R with *Hind*III and *Not*I sites at the 5′ end, and a 3′ tail that overlaps with *meaB*. A 474 bp fragment of *meaB* was amplified with primers KS laeA-meaB F and KS meaB-A-B R. This fragment had a 5′ tail that overlaps with *laeA* and 3′ *Asc*I and *Bam*HI sites. The two fragments were joined by PCR, and the resulting construct was digested with *Asc*I and *Nco*I and ligated into pTMH44.2. This resulted in the plasmid pKJA38.6. The *laeA-meaB* construct was then digested with *Not*I and *Bam*HI and ligated into pKJA38.6 to generate pKJA39.1. *A. parasiticus pyrG* was cut from pJW66.3 [43] using *Eco*RI and then inserted into the *Eco*RI site of pKJA39.1 to create pKJA40.1. PCR was used to confirm each step of construction.

#### Transformation and strain confirmation

Fungal protoplast preparation and transformation were carried out using a polyethylene glycol method [44]. Protoplasts were mixed with 6 µg of the constructed PCR cassette described above. Over-expression transformants were confirmed by PCR using *A. nidulans* gpdAFor and AFOEmeaB3FRev. Southern analysis was used to confirm correct transformants. Probes were created with AFOEmeaB5FfFor and AFOEmeaB3FRev from *A. flavus* NRRL3357 wild type gDNA for Southern analysis. *A. flavus meaB* deletant strains were created by adding 5 µg of the *pyrG* replacement vectors described above with protoplasts of *A. flavus* NRRL3357.5 and plated on minimal medium containing no supplements. The twenty mutants were examined by PCR and further confirmed by Southern analysis with OEAFmeaB5FnestedFor and KOAFmeaB3FnestedRev primers. The *laeA, meaB* double knockdown strain was created by mixing pKJA40.1 with protoplasts of *A. flavus* NRRL3357.5 and plating on minimal medium. Transformants were first confirmed by Northern blot. For this, 10^6^ spores/mL were inoculated into 50 mL GMM and shaken for 24 hours at 225 rpm at 30°C. RNA was extracted using the Trizol method (Invitrogen), and the blots were hybridized with gene fragments amplified by primers KS laeA int F and R for *laeA*, and KS meaB int F and R for *meaB*. All isolates that exhibited the characteristic smear indicating degraded transcript were probed by Southern blot with probe primers KS JP-M13 F and KS JP-M13-R.

### Northern Analysis

Three media with different nitrogen sources were prepared: 1. liquid GMM containing no nitrogen source, 2. liquid GMM using nitrate salts and 3. liquid GMM substituting 10 mM ammonium tartrate for nitrate [39]. Fifty ml of each medium were inoculated with 10^6^ conidia/ml of appropriate strains and incubated with shaking at 250 rpm at 29°C under the light or dark as indicated. After various hours (typically 24 and 48), the mycelium was collected and total RNA was extracted using the Trizol method (Invitrogen). Despite poor growth in the no nitrogen source experiment, the fungi still grew and sufficient mycelium was obtained for Northern analysis. Blots were hybridized with gene fragments amplified from gDNA of NRRL3357 for *A. flavus* using the primers OEAFmeaB3FFor and OEAFmeaB3FRev for *A. flavus meaB*, NAFlaeAFor and NAFlaeARev for *A. flavus laeA*. All other Northern primers were listed on Table 3 as indicated by a N (Northern). Detection of signals was carried out with a Phosphorimager-SI (Molecular Dynamics).

### Pathogenicity Tests

#### Lipase activity

To test for lipase activity, lipase medium (0.5% mycological peptone, 0.3% yeast extract in 1% agar containing 0.1% glyceryl tributyrate) was used in a sterile test tube. Lipase medium was overlaid with 100 µl of 10^6^ conidia/ml for each strain. Tubes were incubated at 29°C in continuous light. Measurements of the clearing zone, indicative of lipase activity, were taken at days 5. Experiment was repeated two times with five biological replications.

#### Seed infections

Mature live peanut seeds (*Arachis hypogaea*) were used to measure pathogenicity of *meaB* mutants in *A flavus* following previously described methods [4]. Briefly, the peanuts were prepared by removing the testa using the fingers after soaking in tap water for 5 min. The two cotyledons of each seed were separated and the embryo carefully removed without damaging the cotyledon tissue. After sterilization, 20 peanut cotyledons were inoculated with 10^5^ spores/cotyledons suspensions of each strain as well as a water control (mock inoculation) and incubated for 30 min in a rotary shaker at 50 rpm. Peanut cotyledons were incubated for 5 days for peanut cv TR96 (harvested from Stephenville, Texas in 2008) at 29°C in dark conditions. The filter paper was moistened daily. All seed experiments were repeated two times with three biological replications.

#### Aflatoxin extraction from seed

Inoculated peanut cotyledons were immediately processed to extract aflatoxin, as described before [4]. The extracts from inoculated peanut cotyledons were dried for three days and then re-suspended in 500 µl of chloroform and 10 µl of each extract was separated on a silica gel TLC plate using the chloroform:acetone (95:5 v/v) solvent system. Extractions were repeated two times with three biological replications.

### Growth on Different Nitrogen Sources

To assess ability of the *meaB* mutants to utilize different nitrogen sources, colony growth was assessed on the following media: GMM containing no nitrogen source, GMM with 10 mM sodium nitrate, GMM with 10 mM ammonium chloride, GMM with 10 mM ammonium chloride plus 30 mM sodium nitrite, GMM with 10 mM ammonium chloride plus 200 mM potassium chlorate, and GMM with 10 mM sodium nitrate plus 100 mM methylammonium chloride [23], [30]. Uracil and uridine were added to all media at 5 mM each. 1 µL containing 10^3^ spores was point inoculated on 40 mL of media and incubated for 3 days at 29°C. The experiment was performed with four replicates and repeated two times.

### Aflatoxin Analysis

Flasks containing 25 mL of either GMM with no nitrogen, standard GMM (70.6 mM sodium nitrate), or GMM with 70.6 mM ammonium chloride were inoculated with 10^6^ conidia/mL of each strain and incubated with shaking at 250 rpm at 29°C. After 48 h, 10 mL of chloroform was added to each flask, and samples were mixed gently at room temperature for 30 minutes. The lower layer (chloroform) was transferred to clean glass vials and allowed to dry for 3 days, then resuspended in 25 to 100 µl of chloroform, and 10 µl of suspension was applied to thin-layer chromatography (TLC) plates (Whatman, Maidstone, England). TLC plates were developed using chloroform:acetone (95:5, vol/vol) solvent system and visualized under 254 nm light. Each strain was grown in duplicate, and the entire experiment was repeated two times.

### Sclerotial Assays

Sclerotial formation was measured for fungal strains following previously described methods [4]. Briefly, 10 ml of GMM media with 1.6% agar and 2% sorbitol was overlaid with 3 ml of GMM media with 0.7% agar and 2% sorbitol containing 10^3^ spores/plate of each *A. flavus* strain. Cultures were grown at 29°C under complete darkness for six days. To visualize sclerotium formation, plates were sprayed with 70% ethanol to kill and wash away conidia. The exposed sclerotia were then collected, lyophilized, and weighed (dry weight per plate). Sclerotial weight was determined by using four replicates.

### Statistical Analysis

Statistical differences were analyzed using the JMP software package (version 9.0.2, SAS Institute, Inc, Cary, NC).

## Supporting Information

Figure S1 Diagram of creation of *meaB* mutants and identification of mutants by Southern analysis. Transformants were screened by at least two different endonucleases. Asterisks show the correct mutants. Bold (deletion and over-expression) and dashed (RNAi silencing knock-down) lines on wild type (WT) locus indicate the radioactive probe sites. (A) Over-expression of *A. flavus meaB* with *A. nidulans gpdA* promoter. N: *Nde*I (5295 bp for WT, 6137 bp and 4304 bp for OE::meaB mutant.) (B) Deletion of *A. flavus meaB*, replaced with the *A. fumigatus pyrG*. S: *Sph*I (4202 bp and 3886 bp for WT, 8414 bp for *ΔmeaB* mutant.) (C) Deletion of *A. flavus meaB*, replaced with *A. parasiticus pyrG*. S: *Sph*I (4202 bp and 3886 bp for WT, 9467 bp for *ΔmeaB* mutant.) (D) Knock-down of *A. flavus meaB* through RNAi technology. X: *Xma*I (3888 bp for WT, 3888 bp and extra copy from RNA silencing plasmid, pSA17.3.).
(TIFF)

Figure S2 *Aspergillus flavus meaB* deletion and KD strains require uracil and uridine supplementation for optimal growth on laboratory medium. Δ*meaB = *gene deletion, *OE::meaB = *over-expression of *meaB*, *KDmeaB* = knock down *meaB*,. +UU = supplementation with uracil and uridine.
(TIFF)

Figure S3 Confirmation of simultaneous depletion of *A. flavus laeA* and *meaB*. (A) Diagram of portion of plasmid pKJA40.1 used to deplete both *A. flavus laeA* and *meaB*. The *A. nidulans* constitutive *gpdA* promoter (*gpdA(p)*) drives expression of inverted copies of *laeA* and *meaB* gene fragments, which are separated by a short spacer. The *A. nidulans trpC* terminator (*trpC(t)*) stops transcription. “S” indicates the location of the *Stu*I site, and the thick black bar above *gpdA(p)* represents where the probe that was used for Southern analysis hybridizes. (B) 10^6^ spores per mL from each of twelve transformants were inoculated into 50 mL GMM and shaken for 30 hours at 250 rpm at 29°C. RNA was extracted and probed by Northern blot with gene fragments corresponding to *laeA* (left blot) and *meaB* (right blot). Ribosomal RNA bands are shown below each blot. Lanes marked with an asterisk indicate transformants that are undergoing degradation of *laeA* and *meaB* based on the smearing pattern. (C) Southern analysis was carried out for correct isolates from (B). DNA was cut with *Stu*I and probed with a fragment corresponding to a portion of the *A. nidulans gpdA(p)* to generate one band per copy of integrated plasmid. The wild type (WT) and parental strain (P, NRRL3357.5) were probed as well and exhibit three faint background bands. Those isolates marked with an asterisk display the same three background bands in addition to at least one other band representing the plasmid.
(TIF)

Figure S4 Figure 4. Northern analysis of steA in *A. flavus meaB* mutants grown for 48 hours in different nitrogen sources. WT = NRRL3357; Δ*meaB* = TSA14.13; *OE::meaB* = TSA15.18. All media was supplemented with uracil and uridine. Ribosomal RNA (rRNA) is shown as the loading control. Probes are written on the left.
(TIF)
