# Supplementary figures and images for: The bZIP Protein MeaB Mediates Virulence Attributes in Aspergillus flavus
Source: PLoS One. 2013 Sep 9;8(9):e74030. doi: 10.1371/journal.pone.0074030 (PMC3767667; doi:10.1371/journal.pone.0074030)

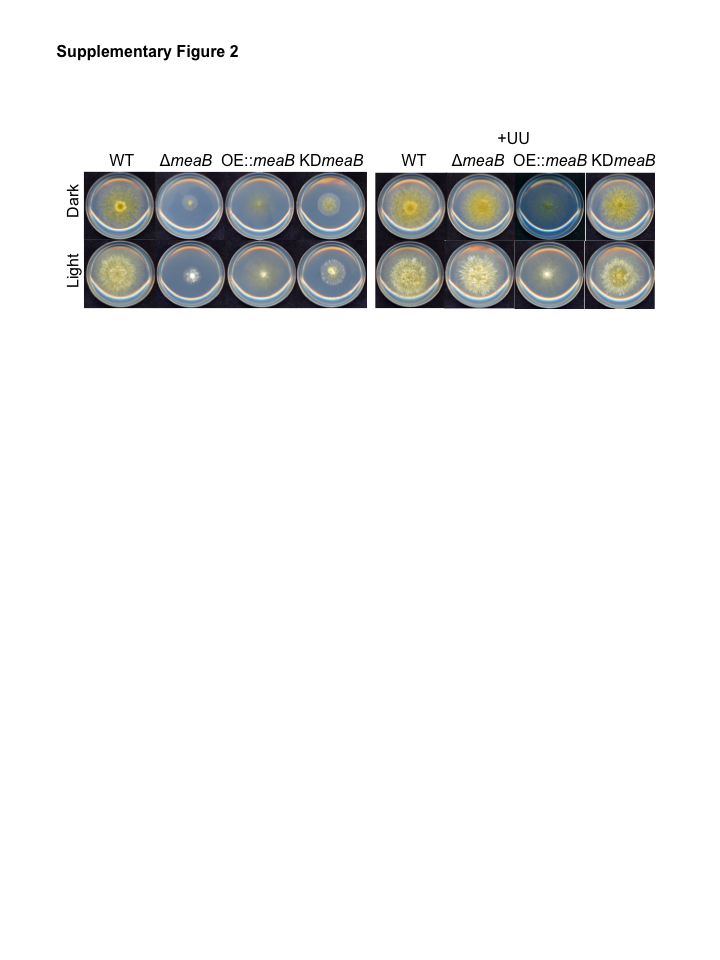

Supplement: Figure S2 — Aspergillus flavus meaB deletion and KD strains require uracil and uridine supplementation for optimal growth on laboratory medium. ΔmeaB = gene deletion, OE::meaB = over-expression of meaB, KDmeaB = knock down meaB,. +UU = supplementation with uracil and uridine. (TIFF) [file pone.0074030.s002.tif]

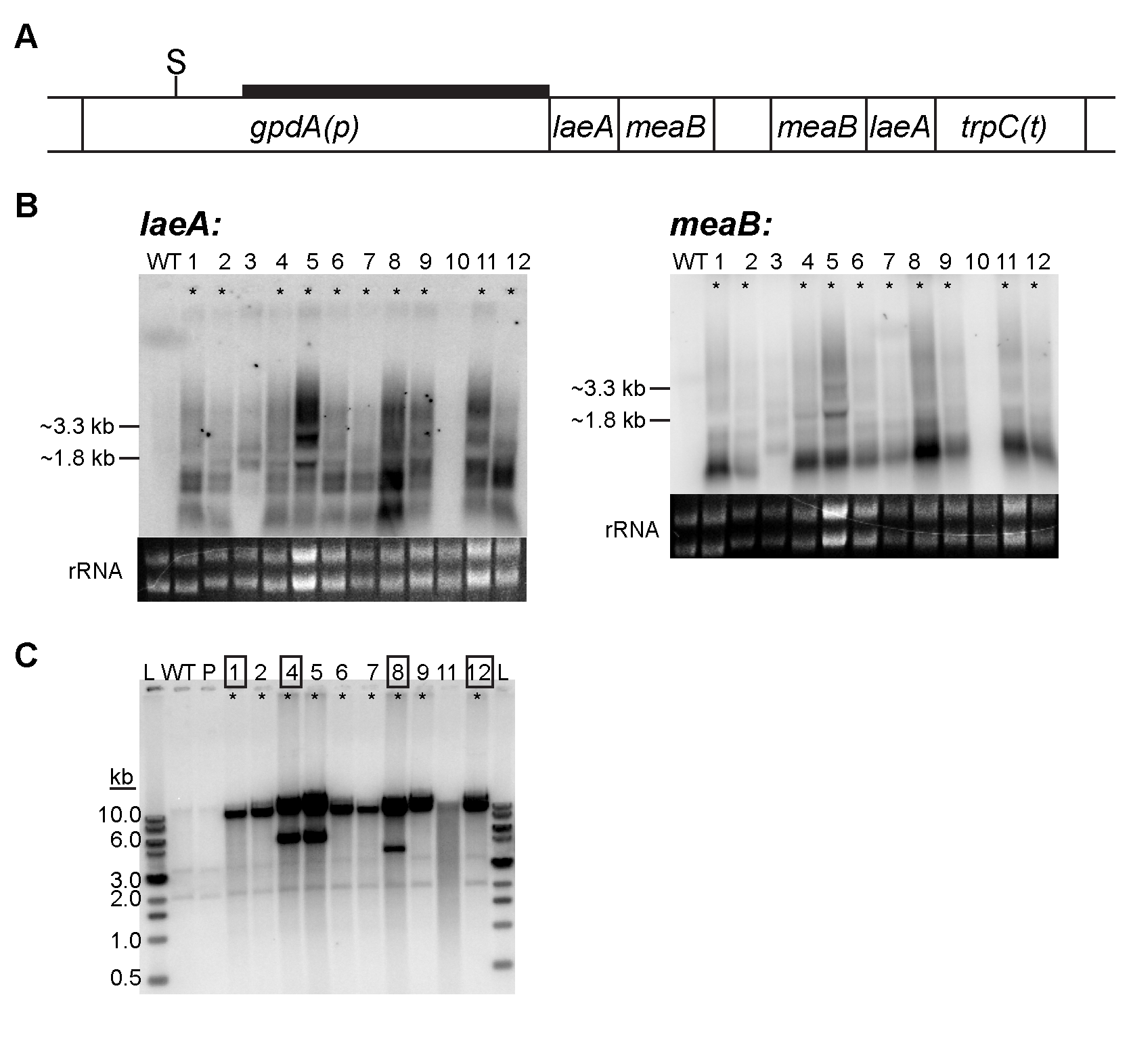

Supplement: Figure S3 — Confirmation of simultaneous depletion of A. flavus laeA and meaB. (A) Diagram of portion of plasmid pKJA40.1 used to deplete both A. flavus laeA and meaB. The A. nidulans constitutive gpdA promoter (gpdA(p)) drives expression of inverted copies of laeA and meaB gene fragments, which are separated by a short spacer. The A. nidulans trpC terminator (trpC(t)) stops transcription. “S” indicates the location of the StuI site, and the thick black bar above gpdA(p) represents where the probe that was used for Southern analysis hybridizes. (B) 106 spores per mL from each of twelve transformants were inoculated into 50 mL GMM and shaken for 30 hours at 250 rpm at 29°C. RNA was extracted and probed by Northern blot with gene fragments corresponding to laeA (left blot) and meaB (right blot). Ribosomal RNA bands are shown below each blot. Lanes marked with an asterisk indicate transformants that are undergoing degradation of laeA and meaB based on the smearing pattern. (C) Southern analysis was carried out for correct isolates from (B). DNA was cut with StuI and probed with a fragment corresponding to a portion of the A. nidulans gpdA(p) to generate one band per copy of integrated plasmid. The wild type (WT) and parental strain (P, NRRL3357.5) were probed as well and exhibit three faint background bands. Those isolates marked with an asterisk display the same three background bands in addition to at least one other band representing the plasmid. (TIF) [file pone.0074030.s003.tif]

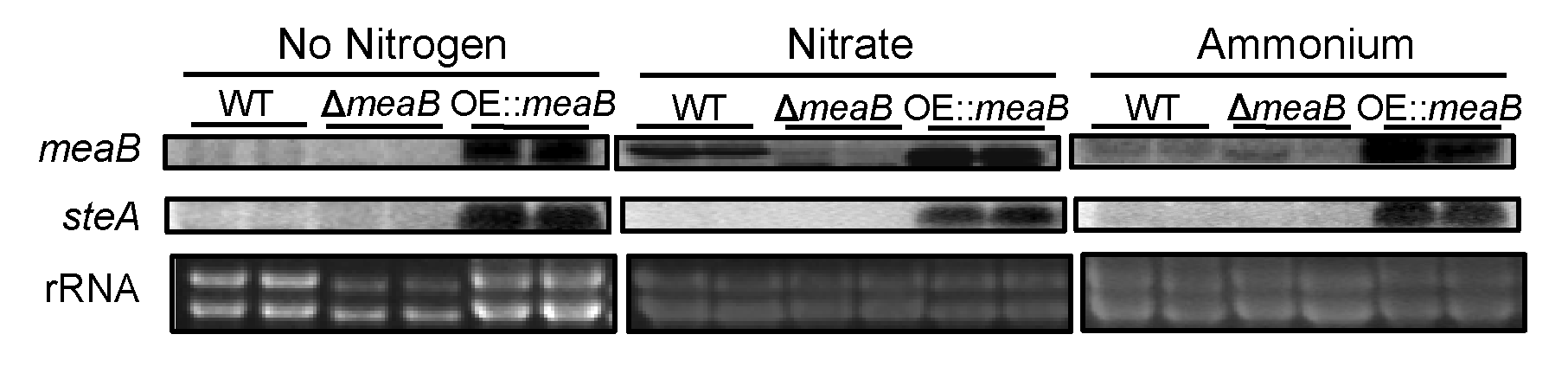

Supplement: Figure S4 — Figure 4. Northern analysis of steA in A. flavus meaB mutants grown for 48 hours in different nitrogen sources. WT = NRRL3357; ΔmeaB = TSA14.13; OE::meaB = TSA15.18. All media was supplemented with uracil and uridine. Ribosomal RNA (rRNA) is shown as the loading control. Probes are written on the left. (TIF) [file pone.0074030.s004.tif]
